# The relationships between social participation before the COVID-19 pandemic and preventive and health-promoting behaviors during the pandemic: the JAGES 2019–2020 longitudinal study

**DOI:** 10.1265/ehpm.22-00154

**Published:** 2022-11-09

**Authors:** Miyako Kimura, Kazushige Ide, Koryu Sato, Eunji Bang, Toshiyuki Ojima, Katsunori Kondo

**Affiliations:** 1 Department of Preventive Medicine, St. Marianna University School of Medicine, 2-16-1, Sugao, Miyamae-ku, Kawasaki-shi, 216-8511, Japan; 2 Department of Social Preventive Medical Sciences, Center for Preventive Medical Sciences, Chiba University, Chiba-shi, Chiba, Japan; 3 Department of Community General Support, Hasegawa Hospital, Yachimata, Chiba, Japan; 4 Department of Social Epidemiology, Graduate School of Medicine and School of Public Health, Kyoto University, Kyoto-shi, Kyoto, Japan; 5 Japan Agency for Gerontological Evaluation Study (JAGES Agency), Kashiwa-shi, Chiba, Japan; 6 Hamamatsu University School of Medicine, Department of Community Health & Preventive Medicine, Hamamatsu-shi, Shizuoka, Japan; 7 Center for Gerontology and Social Science, Research Institute, National Center for Geriatrics and Gerontology, Obu-shi, Aichi, Japan

**Keywords:** COVID-19, Health-promoting behaviors, Older people, Preventive behavior, Social participation

## Abstract

**Background:**

People’s preventive behavior is crucial for reducing the infection and transmission of a novel coronavirus, especially in aging societies. Moreover, since behavioral restrictions may lead to high risks of secondary health impacts among older people, health-promoting behaviors, including proper nutrition intake and regular exercise, should also be encouraged. Although various studies have reported the positive association between social participation and health among older people, whether their social participation relates to preventive and health-promoting behaviors during the COVID-19 pandemic was uncertain. This study examined the relationships between social participation before the COVID-19 pandemic and preventive and health-promoting behaviors during the pandemic among older people in Japan.

**Methods:**

We obtained longitudinal data from the Japan Gerontological Evaluation Study (JAGES), which conducted baseline and follow-up surveys from November 2019 to January 2020 (pre-pandemic) and from November 2020 to February 2021 (during the pandemic) in ten municipalities. In total, 10,523 responses were analyzed. Preventive and health-promoting behaviors were measured by nine actions (e.g., wash/disinfect hands, wear masks, do exercise), and the total of these actions was divided into two (highly implemented ≥7 or not highly implemented <7). Social participation was assessed by nine activities (e.g., participating in volunteering, sports clubs, had paid work). Adjusted for covariates, we examined the relationships between each social participation and preventive and health-promoting behavior by the logistic regression analysis or the Poisson regression analysis.

**Results:**

Older people who participated in social activities pre-pandemic showed a tendency to implement preventive and health-promoting behaviors during the pandemic. Especially, participations in “sports” and “Kayoi-no-ba” were positively related to “do exercise.” Only “had paid work” was negatively related to highly implemented preventive and health-promoting behaviors.

**Conclusions:**

There were the positive relationships between social participation and preventive and health-promoting behavior. This study also indicated that older people who did not participate in social activities or had paid work before the COVID-19 pandemic may have higher risks of infection and secondary health impacts. Taking into account such old people’s lifestyles as well as their workplace conditions, promoting appropriate behaviors need to be considered.

## Graphical Abstract

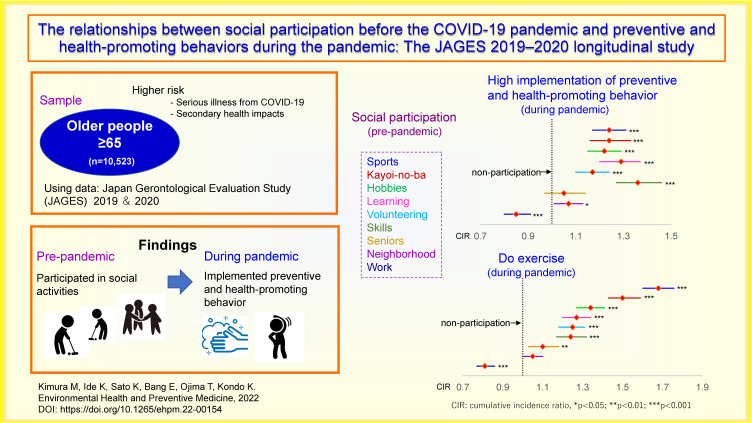

## Background

By February 18, 2022, COVID-19 had afflicted approximately 400 million people and killed approximately 5.8 million people worldwide [1]. Two years have passed since the World Health Organization (WHO) declared COVID-19 a pandemic on March 11, 2020 [2], yet waves of infection continue to threaten human health and lives [1].

Although Japan has avoided a lockdown to date, unprecedented measures were adopted during the first wave when vaccines had not yet been available. On April 7, 2020, the Head of the Novel Coronavirus Response Headquarters in Japan declared the first national state of emergency. The government requested citizens of the 13 prefectures (Tokyo, Osaka, Hokkaido, Ibaraki, Saitama, Chiba, Kanagawa, Ishikawa, Gifu, Aichi, Kyoto, Hyogo, and Fukuoka) be designated as “Prefectures under Specific Cautions,” to refrain from leaving home, moving to other prefectures, or visiting downtown restaurants with hospitality services [3]. The government has recommended avoidance of the three Cs to citizens: closed spaces, crowded spaces, and close-contact settings [3]. Thus, people’s preventive behavior has become crucial, both for their own health and for society.

In order to consider effective approaches to encourage preventive behaviors, relevant factors of these behaviors need to be clarified. Previous studies reported that demographics (e.g. older people, females, people with higher education, married/cohabitating), knowledge, perceptions (e.g. effectiveness/benefits of preventive behaviors, risks of infections) and other psychological and social factors (e.g. self-efficacy, fear, anxiety, regret, altruism, conformity, social/subjective norms) were related to preventive behavior during the COVID-19 pandemic [4–9]. Most findings of these were underpinned by theoretical frameworks such as the health belief model (HBM) [5, 10] and the theory of planned behavior (TPB) [8, 11].

Moreover, group behaviors may shape individual behaviors. According to independence theory, when individuals perceive themselves as members of a group, the group’s goals become their own goals [12]. Indeed, a study reported that a sense of belonging to one’s family promoted preventive behavior intentions during the COVID-19 pandemic [13]. Furthermore, since there is a tendency that people are concerned about being accepted by members of their own group [14], participation in group may be related to preventive behaviors.

Similarly, to reduce secondary health impacts among older people, health-promoting behaviors (e.g., proper nutrition intake and regular exercise) [15] have also been recommended. Secondary impacts have been defined as the indirect effects caused by the COVID-19 pandemic [16], which may occur in three main ways; (1) malnutrition and weight loss, (2) physical deconditioning, and (3) psycho-social impacts [17]. Therefore, although behavior restrictions are necessary to reduce the risk of infection, avoiding secondary impacts on older people should be considered [18, 19]. In fact, since Japan has the highest proportion of older people in the world (28.1% in 2018) [20], the government has strongly recommended older people to do exercise and eat well-balanced meals during the pandemic [21].

Various studies have reported the positive association between social participation and health among older people [18, 19], and some studies also presented the positive relations between social participation and fruit/vegetable intake, eating behavior, drinking, and exercise [22]. These results indicated that older people who participated in social activities might engage in desirable health-promoting behavior during the pandemic, compared with those who did not. However, to the best of the authors’ knowledge, no longitudinal study has explored how social participation before the COVID-19 pandemic related to preventive and health-promoting behaviors during the COVID-19 pandemic.

Therefore, this study examined the relationships between social participation before the COVID-19 pandemic and preventive and health-promoting behaviors during the pandemic among older people in Japan.

## Methods

### Data collection and participants

We obtained panel data from the Japan Gerontological Evaluation Study (JAGES), which has targeted people aged 65 or older in Japan. The JAGES is a population-based gerontological study focusing on the social determinants of health and the social environment among older functionally independent people in Japan [23, 24]. A baseline survey was conducted from November 2019 to January 2020, and ten municipalities participated in the study [25]. A total of 88,476 self-reported questionnaires were sent to eligible residents using random sampling, and 62,973 questionnaires were returned (the response rate was 71.2%). From November 2020 to February 2021, a follow-up survey was conducted, and a total of 12,705 questionnaires were sent to the randomly selected participants who participated in the baseline survey. Among the invited residents, 10,860 returned the questionnaires; the response rate of the follow-up survey was 85.5%.

After excluding participants whose sex and age could not be confirmed, 10,523 responses were analyzed. We obtained permission from the JAGES investigators to use the data, which were approved by ethics committees at the National Center for Geriatrics and Gerontology (1274-2) and Chiba University (3442). All participants were asked for their written consent when completing the questionnaire and returned it to us.

### Dependent variables

We examined the participants' preventive and health-promoting behaviors from April to May 2020, and asked whether they implemented the following actions: 1) wash hands frequently and disinfect hands with alcohol (wash/disinfect hands); 2) wear a mask when going out (wear masks); 3) maintain a distance of at least 2 m from others (keep social distance); 4) ventilate or sanitize the room frequently (ventilate/sanitize rooms); 5) following cough etiquette (demonstrate cough etiquette); 6) stretch and exercise (do exercise); 7) avoid places where people gather (avoid crowded places); 8) eat a nutritious diet (eat healthy meals), and 9) recommend others to refrain from going out and wear masks when outdoors (recommend preventive behaviors). Based on the recommendations of the Japanese government and previous studies [5, 21, 26], these variables were used to assess preventive and health-promoting behaviors. First, each behavior was divided into two parts: implemented = 1, or not implemented = 0. Next, those nine items were added up, and the total score was divided into three groups (low, medium, and high) to have approximately equal percentages. Then, the high score group was categorized as the group with highly implemented preventive and health-promoting behavior (≥7), and the medium and low score groups were categorized as the group with not highly implemented such behavior (<7). To examine the appropriateness of this cutoff point, we also performed a sensitivity analysis for cutoff points 6 and 8.

### Independent variables

Social participation of older people has been assessed by the participation in community organizations [27], which has been recommended by the Japanese government as a care prevention measure [28]. Thus, based on previous studies and surveys [27–29] this study used the following types of social participation: volunteer groups (volunteering), sports groups/clubs (sports), hobbies groups (hobbies), senior citizen clubs (seniors), neighborhood associations (neighborhood), learning/cultural groups (learning), community gathering “Kayoi-no-ba” (Kayoi-no-ba), activities to teach skills/pass on experiences to others (skills), and had paid work (work). Community gathering “Kayoi-no-ba,” a mutual focal point, and locally living older people can work on health promotion through various activities in this place [30]. In addition, to promote social activities and build social capital for the prevention of functional disability, “Kayoi-no-ba” has been supported by the Japanese government and local governments [31, 32]. The degree of social participation at the time of the baseline survey (November 2019 to January 2020) was measured with the following questions: “How often do you participate in the following clubs or groups?” Participants were asked to select one answer from the following options: “Almost every day,” “A few times a week,” “Once a week,” “Once or twice a month,” “A few times a year,” or “Never.” Responses were categorized as “yes” if the respondent chose one of the options among “almost every day,” “a few times a week,” “once a week,” “once or twice a month,” and “a few times a year,” and “no” if the choice was “never.” Each social participation was treated as a binary variable (yes = 1, no = 0).

### Covariates

Based on the previous study [25], we adjusted for age, sex, marital status (married or not married, including widowed, divorced, never married), education (less than 10 years or longer), annual equalized household income (low [≤1.9], middle [2.0–3.9], and high [≥4.0] million Japanese Yen [JPY; $1 US = approximately JPY 115 in February 2022]), living status (living alone or living with others), self-reported medical illness (having preexisting illness or not), living areas (living in one of 13 designated prefectures under Specific Cautions and population ≥100,000 or others), and instrumental activities of daily living that was assessed by the Tokyo Metropolitan Institute of Gerontology Index of Competence (independent [5points], or dependent [≤4]) [25, 33]. Depressive symptoms were assessed by the 15-item Geriatric Depression Scale (GDS), and the total score was divided into two (had depressive symptoms [5≥] or not [5<]) [25, 34].

### Statistics

A multivariate analysis was performed with each preventive and health-promoting behavior as the dependent variable. Adjusted for covariates, we examined the relationships between social participation and preventive and health-promoting behavior. Different methods were used depending on the nature of the outcomes. When outcomes were greater than 10%, there was a possibility that the relative risk would be overestimated in the calculation of odds ratios according to logistic regression analysis [35]. Thus, we performed the Poisson regression analysis for common binary outcomes (incidence: 10–90%), and the logistic regression analysis for rare binary outcomes (incidence: <10% or >90%). We applied Robust error estimation to deal with the overestimation of variance in applying Poisson regression to binary data [36]. We categorized missing values of covariates as missing, and performed missing indicator analysis. Even in the presence of missing values of GDS, cases in which the presence or absence of depression could be determined were included in the analysis. All cases that could not be determined were categorized as its missing values. STATA V15 (Stata Corp, College Station, TX, USA) was used for the analyses, a p-value of 0.05 (two-tailed tests) set as the significance level.

## Results

Characteristics of the participants are shown in Table 1. Descriptive statistics of social participation and preventive and health-promoting behavior are presented in Table 2. Higher implementation rates of preventive and health-promoting behaviors were found for “wear masks” (96.8%), “wash/disinfect hands” (90.6%), and “avoid crowded places” (77.8%). Additionally, 33.8% of the participants were categorized as highly implemented preventive and health-promoting behaviors”—that is, implementing seven or more preventive and health-promoting behaviors out of nine. The relationships between social participation and preventive and health-promoting behaviors are shown in Table 3.

**Table 1:** Characteristics of participants (n = 10,523)

	**N**	**%**
Sex		
Male	5,269	50.1
Female	5,254	49.9
Age		
65–74	5,309	50.5
75+	5,214	49.6
Education		
Less than 10	2,122	20.2
10+	8,185	77.8
Missing	216	2.1
Household income		
Low	4,081	38.8
Middle	4,052	38.5
High	1,372	13.0
Missing	1,018	9.7
Marital status		
Married	7,528	71.5
Not married	2,828	26.9
Missing	167	1.6
Living status		
Living alone	1,678	16.0
Living with others	8,261	78.5
Missing	584	5.5
Self-reported medical illness		
Having preexisting illness	8,037	76.4
Not having preexisting illness	1,975	18.8
Missing	511	4.9
Independence of IADL		
Independent	9,555	90.8
dependent	730	6.9
Missing	238	2.3
Living areas		
Living in 13 designated prefectures & population ≥100,000	8,870	84.3
Others	1,653	15.7
Depressive symptoms		
Had depressive symptoms	2,124	20.2
Not had depressive symptoms	7,898	75.1
Missing	501	4.8

IADL: Instrumental Activities of Daily Living

**Table 2:** Social participation (2019) and preventive health behavior (2020) (n = 10,523)

	**N**	**%**		**N**	**%**
	
Social participation			Preventive and health-promoting behavior		
Volunteering			Wash/disinfect hands		
Yes	2,018	19.2	Implemented	9,535	90.6
No	7,036	66.9	Not implemented	988	9.4
Missing	1,469	14.0	Wear masks		
Sports			Implemented	10,190	96.8
Yes	3,220	30.6	Not implemented	333	3.2
No	5,912	56.2	Keep social distance		
Missing	1,391	13.2	Implemented	5,462	51.9
Hobbies			Not implemented	5,061	48.1
Yes	4,286	40.7	Ventilate/sanitize rooms		
No	4,994	47.5	Implemented	5,435	51.7
Missing	1,243	11.8	Not implemented	5,088	48.4
Seniors			Do cough etiquette		
Yes	1,185	11.3	Implemented	6,294	59.8
No	7,978	75.8	Not implemented	4,229	40.2
Missing	1,360	12.9	Do exercise		
Neighborhood			Implemented	4,307	40.9
Yes	3,319	31.5	Not implemented	6,216	59.1
No	5,884	55.9	Avoid crowded places		
Missing	1,320	12.5	Implemented	8,190	77.8
Learning			Not implemented	2,333	22.2
Yes	1,436	13.6	Eat healthy meals		
No	7,633	72.5	Implemented	5,368	51.0
Missing	1,454	13.8	Not implemented	5,155	49.0
Kayoi-no-ba			Recommend preventive behavior		
Yes	1,415	13.5	Implemented	2,980	28.3
No	7,793	74.1	Not implemented	7,543	71.7
Missing	1,315	12.5	Implement preventive and health-promoting behavior (total)		
Skills			Highly implemented (≥7)	3,553	33.8
Yes	1,107	10.5	Not highly implemented (<7)	6,970	66.2
No	8,038	76.4			
Missing	1,378	13.1			
Work					
Yes	3,189	30.3			
No	6,512	61.9			
Missing	822	7.8			

**Table 3:** The relationships between social participation (2019) and preventive and health-promoting promoting behaviors (2020)

**Preventive and health-promoting behavior in 2020**	**Wash/disinfects hands**				**Wear masks**				**Keep social distance**				**Ventilate/sanitize rooms**				**Do cough etiquette**			
				
**Social participation in 2019**	**OR**	**[95%CI]**		**P**	**OR**	**[95%CI]**		**P**	**CIR**	**[95%CI]**		**P**	**CIR**	**[95%CI]**		**P**	**CIR**	**[95%CI]**		**P**
					
Volunteering n = 9,054	1.22	1.01	1.48	0.040	1.05	0.77	1.43	0.749	1.06	1.01	1.11	0.016	1.07	1.03	1.12	0.002	1.05	1.01	1.09	0.009
Sports n = 9,132	1.39	1.17	1.64	<0.001	1.10	0.83	1.45	0.499	1.00	0.96	1.04	0.886	1.00	0.96	1.04	0.994	1.04	1.00	1.07	0.025
Hobbies n = 9,280	1.22	1.05	1.42	0.011	1.01	0.78	1.30	0.947	1.01	0.97	1.05	0.590	1.02	0.98	1.07	0.234	1.06	1.02	1.09	0.001
Seniors n = 9,163	1.02	0.82	1.27	0.849	1.14	0.79	1.66	0.489	0.98	0.92	1.04	0.547	1.02	0.96	1.08	0.567	1.01	0.96	1.06	0.654
Neighborhood n = 9,203	1.11	0.95	1.30	0.180	1.23	0.94	1.60	0.128	1.03	0.99	1.07	0.188	1.07	1.02	1.11	0.002	1.02	0.99	1.06	0.170
Learning n = 9,069	1.27	1.00	1.60	0.046	1.50	0.98	2.29	0.062	1.11	1.06	1.17	<0.001	1.08	1.03	1.14	0.001	1.08	1.03	1.12	<0.001
Kayoi-no-ba n = 9,208	1.11	0.89	1.39	0.359	0.96	0.67	1.39	0.840	1.04	0.98	1.09	0.202	1.07	1.02	1.12	0.008	1.01	0.97	1.06	0.641
Skills n = 9,145	1.29	1.01	1.67	0.045	0.98	0.66	1.44	0.901	1.10	1.05	1.17	<0.001	1.16	1.10	1.22	<0.001	1.10	1.06	1.15	<0.001
Work n = 9,701	1.11	0.94	1.30	0.221	0.97	0.74	1.27	0.840	0.89	0.86	0.93	<0.001	0.99	0.95	1.04	0.819	0.99	0.95	1.02	0.459

**Preventive and health-promoting behavior in 2020**	**Do exercise**				**Avoid crowded places**				**Eat healthy meals**				**Recommend preventive behavior**				**Highly implemented preventive and health-promoting behavior**			
				
**Social participation in 2019**	**CIR**	**[95%CI]**		**P**	**CIR**	**[95%CI]**		**P**	**CIR**	**[95%CI]**		**P**	**CIR**	**[95%CI]**		**P**	**CIR**	**[95%CI]**		**P**
					
Volunteering n = 9,054	1.25	1.18	1.31	<0.001	0.99	0.97	1.02	0.673	1.10	1.05	1.15	0.001	1.15	1.06	1.24	<0.001	1.17	1.10	1.24	<0.001
Sports n = 9,132	1.68	1.60	1.76	<0.001	0.97	0.95	0.99	0.005	1.12	1.07	1.16	<0.001	1.05	0.97	1.12	0.212	1.24	1.17	1.31	<0.001
Hobbies n = 9,280	1.34	1.27	1.41	<0.001	0.99	0.97	1.01	0.314	1.15	1.10	1.20	<0.001	1.13	1.06	1.21	<0.001	1.22	1.15	1.29	<0.001
Seniors n = 9,163	1.10	1.03	1.18	0.004	0.98	0.95	1.02	0.353	1.07	1.02	1.13	0.008	1.21	1.10	1.32	<0.001	1.05	0.97	1.14	0.214
Neighborhood n = 9,203	1.05	1.00	1.10	0.078	1.00	0.98	1.03	0.767	1.03	0.99	1.07	0.193	1.10	1.03	1.17	0.007	1.07	1.01	1.13	0.026
Learning n = 9,069	1.27	1.20	1.34	<0.001	1.03	1.00	1.06	0.058	1.21	1.16	1.27	<0.001	1.14	1.05	1.25	0.002	1.29	1.20	1.37	<0.001
Kayoi-no-ba n = 9,208	1.50	1.43	1.59	<0.001	0.98	0.95	1.01	0.264	1.13	1.08	1.18	<0.001	1.18	1.09	1.28	<0.001	1.24	1.16	1.33	<0.001
Skills n = 9,145	1.24	1.17	1.32	<0.001	1.00	0.97	1.03	0.882	1.21	1.15	1.27	<0.001	1.30	1.19	1.42	<0.001	1.36	1.27	1.46	<0.001
Work n = 9,701	0.81	0.77	0.86	<0.001	0.95	0.92	0.97	<0.001	0.93	0.89	0.97	0.001	1.02	0.94	1.09	0.669	0.85	0.80	0.91	<0.001

Adjusted for age, sex, marital status, annual equalized household income, education, living status, self-reported medical illness, living in 13 designated prefectures and population ≥100,000, instrumental activities of daily living, depressive symptoms. Ref. non-participation in the respective social activity in 2019.

OR: odds ratios, CIR: cumulative incidence ratio, CI: confidence interval

Compared to the non-participating group, seven out of the nine types of social participation groups showed positive relation to highly implement preventive and health-promoting behavior. Moreover, seven types of social participation were positively related to “do exercise” and “eat healthy meals.” Especially, participation in “sports” (cumulative incidence ratio: CIR = 1.68, 95% CI = 1.60–1.76, p < 0.001) and “Kayoi-no-ba” (CIR = 1.50, 95% CI = 1.43–1.59, p < 0.001) were positively related to “do exercise.”

Only “had paid work” was negatively related to preventive and health-promoting behaviors (keep social distance: CIR = 0.89, 95% CI = 0.86–0.93, p < 0.001; do exercise: CIR = 0.81, 95% CI = 0.77–0.86, p < 0.001; avoid crowded places: CIR = 0.95, 95% CI = 0.92–0.97, p < 0.001; eat healthy meals: CIR = 0.93, 95% CI = 0.89–0.97, p = 0.001; and highly implemented preventive and health-promoting behaviors CIR = 0.85, 95% CI = 0.80–0.91, p < 0.001).

We performed the sensitivity analyses wherein the cutoff points for highly implementing preventive and health-promoting behaviors were set to 8 or more and 6 or fewer points. Then, we confirmed that at three different cutoff points (6,7,8), there were no differences in the positive or negative associations between each social participation and preventive and health-promoting behaviors.

## Discussion

This is the first study to examine the relationships between social participation before the COVID-19 pandemic and preventive and health-promoting behaviors during the pandemic. Older people who participated in social activities pre-pandemic showed a tendency to highly implement preventive and health-promoting behaviors during the pandemic. Especially, participation in “sports” and “Kayoi-no-ba” before the pandemic were positively related to “do exercise” during the pandemic. Previous studies showed that older people who participated in “Kayoi-no-ba” were able to obtain a lot of information related to health, food, and exercise [37, 38] and these advantages may have been reflected in the preferable behavior during the pandemic. Since such health-promoting behavior may reduce the risks of secondary health impacts among older people, encouraging social participation for older people during ordinary days can be considered beneficial for their health during an emergency, such as the pandemic.

On the other hand, older people who did not participate in social activities before the pandemic could be considered as having higher risks of infection and secondary health impacts during and after the pandemic. Since older people who did not participate in social activities may have fewer opportunities to obtain health-related information and are less sensitive to their own and others’ health compared to those who participated in social activities, it is necessary to raise their awareness and disseminate information about appropriate behavior. There are many useful examples of all kinds of efforts being made in each municipality in Japan to protect health of older people. For example, a city enclosed a leaflet recommending exercise when sending residents an application for special benefits [39]. Another example is that, considering the typical living conditions of older people who visit post offices to withdraw their pensions, a community has made stockpiles available for purchase at the post office [39]. These approaches may allow older people, who are less active and have fewer opportunities to exchange information with others, to obtain health-related information and foods equally well. Furthermore, as of August 5, 2022, 858 exercise programs for older people are produced by 426 local governments in Japan that can be seen on the web [40], and in some areas, desirable exercises and infection prevention behaviors are broadcast on local television [39]. Although we were unable to clarify outcomes when this study was conducted, it is expected that such approaches might have a positive impact on the behavior of older people without social participation in the medium to long term.

Only one type of participation—“had paid work”—showed a negative association with highly implemented preventive and health-promoting behaviors. The reasons may be that they had more opportunities to work during the COVID-19 pandemic, which made it difficult to maintain distance from people, or they lacked time or mental capacity to pay attention to diet and exercise. Unfortunately, we were unable to identify the social norms and countermeasures toward COVID-19 at their workplaces, but our findings suggest that employed older people before the pandemic might be at a higher risk of COVID-19 infection during the pandemic. Therefore, it is necessary to promote infection prevention not only among older workers, but also in their workplaces.

No association was found between mask use and social participation. This may be because of the high rate of mask use among Japanese people in general, regardless of whether they have social participation—in fact, 96.8% of the participants in this study wore masks. We previously explored preventive health behaviors among mothers of infants and/or preschoolers in Japan, and reported high implementation of “wearing face masks” (87.9%), “washing hands” (88.3%), and “avoiding crowded places” (84.4%) [41]; however, older people in this study also showed the high implementation of these behaviors. Thus, such preventive and health-promoting behaviors may be a characteristic of Japanese people. According to Nakayachi et al. [7], conformity to social norms was found to be the strongest motivator for mask use among Japanese people during the COVID-19 pandemic. However, social norms occasionally lead to a negative impact on group members, such as “the black sheep effect,” which states that in-group deviants tend to be judged more negatively than out-group counterparts [42]. Therefore, group leaders of social activities are expected to prevent this kind of exclusion.

Investigating both positive and negative aspects of social participation may be helpful from the perspective of older people who are unable to adopt preventive and health-promoting behaviors.

Changes in social participation and preventive and health-promoting behavior before/during the COVID-19 pandemic and their impact on health should also be examined in longitudinal studies.

### Limitations

This study has several limitations. Although there were various statistically significant relationships between each social participation and preventive and health-promoting behavior, most of their effect sizes were minimal, and the results might be reflected by the large sample size. In addition, we found that “had paid work” before the pandemic was related to poorer preventive and health-promoting behaviors during the pandemic; however, we were unable to identify what work was related to poor preventive and health-promoting behaviors. Moreover, various psychological factors may affect human behavior, but we could not investigate the relationships between such variables and preventive and health-promoting behaviors in the current study. Furthermore, since we focused on how pre-pandemic social participation relates to preventive and health-promoting behaviors during the pandemic, we did not examine the possible effects of reduced social participation and various other factors during the pandemic.

## Conclusion

Older people who participated in social activities before the COVID-19 pandemic showed a tendency to highly implement preventive and health-promoting behaviors during the pandemic. Our findings also indicated that older people who did not participate in social activities or had paid work may have higher risks of infection and secondary health impacts. Thus, taking into account their lifestyles and workplace conditions, effective ways for promoting appropriate behaviors need to be considered. Future study is needed to examine the changes in social participation and preventive and health-promoting behavior before/during the COVID-19 and their impact on health in the long term.

## Declarations

### Availability of data and materials

No data and materials are available.
